# Diet-induced Weight Loss and Phenotypic Flexibility Among Healthy Overweight Adults: A Randomized Trial

**DOI:** 10.1016/j.ajcnut.2023.07.002

**Published:** 2023-08-28

**Authors:** Milena Rundle, Jarlei Fiamoncini, E Louise Thomas, Suzan Wopereis, Lydia A. Afman, Lorraine Brennan, Christian A. Drevon, Thomas E. Gundersen, Hannelore Daniel, Isabel Garcia Perez, Joram M. Posma, Diana G. Ivanova, Jimmy D. Bell, Ben van Ommen, Gary Frost

**Affiliations:** 1 Section of Nutrition, Department of Metabolism, Digestion and Reproduction, Faculty of Medicine, Imperial College London, London, United Kingdom; 2 Food Research Center, Department of Food Science and Experimental Nutrition, School of Pharmaceutical Sciences, University of São Paulo, São Paulo, Brazil; 3 Research Centre for Optimal Health, School of Life Sciences, University of Westminster, London, United Kingdom; 4 Department of Microbiology and Systems Biology, Netherlands Organization for Applied Scientific Research, Hague, The Netherlands; 5 Division of Human Nutrition and Health, Wageningen University, Wageningen, The Netherlands; 6 UCD School of Agriculture and Food Science, Institute of Food and Health, University College Dublin, Belfield, Dublin, Ireland; 7 Department of Nutrition, Institute of Basic Medical Sciences, Faculty of Medicine, University of Oslo, Oslo, Norway; 8 Vitas Ltd, Oslo Science Park, Oslo, Norway; 9 Hannelore Daniel, Molecular Nutrition Unit, Technische Universität München, München, Germany; 10 Section of Bioinformatics, Department of Metabolism, Digestion and Reproduction, Faculty of Medicine, Imperial College London, London, United Kingdom; 11 Department of Biochemistry, Molecular Medicine and Nutrigenomics, Faculty of Pharmacy, Medical University, Varna, Bulgaria

**Keywords:** phenotypic flexibility, weight loss, insulin sensitivity, meal challenges, metabolites

## Abstract

**Background:**

The capacity of an individual to respond to changes in food intake so that postprandial metabolic perturbations are resolved, and metabolism returns to its pre-prandial state, is called phenotypic flexibility. This ability may be a more important indicator of current health status than metabolic markers in a fasting state.

**Aim:**

In this parallel randomized controlled trial study, an energy-restricted healthy diet and 2 dietary challenges were used to assess the effect of weight loss on phenotypic flexibility.

**Methods:**

Seventy-two volunteers with overweight and obesity underwent a 12-wk dietary intervention. The participants were randomized to a weight loss group (WLG) with 20% less energy intake or a weight-maintenance group (WMG). At weeks 1 and 12, participants were assessed for body composition by MRI. Concurrently, markers of metabolism and insulin sensitivity were obtained from the analysis of plasma metabolome during 2 different dietary challenges—an oral glucose tolerance test (OGTT) and a mixed-meal tolerance test.

**Results:**

Intended weight loss was achieved in the WLG (−5.6 kg, *P <* 0.0001) and induced a significant reduction in total and regional adipose tissue as well as ectopic fat in the liver. Amino acid-based markers of insulin action and resistance such as leucine and glutamate were reduced in the postprandial phase of the OGTT in the WLG by 11.5% and 28%, respectively, after body weight reduction. Weight loss correlated with the magnitude of changes in metabolic responses to dietary challenges. Large interindividual variation in metabolic responses to weight loss was observed.

**Conclusion:**

Application of dietary challenges increased sensitivity to detect metabolic response to weight loss intervention. Large interindividual variation was observed across a wide range of measurements allowing the identification of distinct responses to the weight loss intervention and mechanistic insight into the metabolic response to weight loss.

## Introduction

The maintenance of energy homeostasis in postprandial and postabsorptive periods requires different metabolic processes to be activated either to store excess energy from food intake or to mobilize stored substrates. This dynamic shift between negative and positive energy balance with a transfer of molecules between tissues and regulation of metabolic pathways, is to a large extent coordinated by the dynamic interplay between different hormones such as insulin, glucagon, cortisol, and incretins. The sensitivity of tissues to insulin is very important for regulating the metabolism of carbohydrates, fatty acids, and amino acids. Insulin-dependent regulation of metabolism involves multiple organs, signaling pathways, and metabolites derived from all 3 macronutrients [1]. The concentrations of these metabolites and signaling molecules in biofluids constitute dynamic phenotypic traits.

Although insulin sensitivity has become a marker of overall metabolic health, it does not describe how the metabolic networks respond to particular cues. To achieve homeostasis, physiology maintains a well-orchestrated machinery allowing the organism to adapt to the continuously changing environment, in which food plays a major role [1]. The ability to respond adequately to transient changes in substrate availability induced by food intake may predict the health status of the organism [2,3]. Homeostasis might render relatively insensitive biomarkers in biofluids that are being sampled in the absence of stressors (i.e., after overnight fasting). On the contrary, assessing the same biomarkers in a challenged state might provide more sensitive metabolic information [1,3,4]. As an example, in the absence of a challenging stimulus plasma glucose concentration is tightly regulated by several mechanisms to maintain its levels within a narrow range. Changes in plasma glucose concentrations induced by a meal may allow the detection of early problems in glucose homeostasis because the meal promotes metabolic changes involved in restoring preprandial plasma glucose concentrations [5]. Phenotypic flexibility refers to the response capacity to environmental cues triggering changes in plasma levels of metabolites and signaling molecules.

The NutriTech project aimed to integrate emerging and established technologies to develop a deeper understanding of phenotypic flexibility. In this study, we used a weight loss intervention coupled with a healthy diet in an overweight cohort—an established methodology that has been shown to have a positive effect on insulin sensitivity and phenotypic flexibility [6]. We used 2 dietary challenges to assess phenotypic flexibility: an OGTT and a mixed-meal tolerance test (MMTT), followed by a thorough characterization of body composition and plasma metabolites.

## Subjects and Methods

### Recruitment and study population

Research ethics was granted by the West London Ethic Committee (12/LO/0139) and the study is registered at ClinicalTrials.gov NCT01684917. The **Supplementary Methods** section provides information on recruitment, the screening process, and inclusion and exclusion criteria. All participants provided written, informed consent at the screening visit. The participants were recruited between June 2012 and July 2014 and the intervention run between July 2012 and October 2014. The primary outcome of the study was a change in insulin sensitivity. In total, 72 subjects completed the study. Recruitment numbers and flow are presented in Supplemental Figure 1.

### Dietary intervention study

A randomized comparison of a 20% energy-restricted diet for 12 wk compared with a 12-wk weight-maintenance diet (based on average energy intake in the EU), was conducted in a cohort of adults classified as overweight and obese (average BMI: 29.2; range: 24.7–35.6). Inclusion and exclusion criteria are listed in Supplemental Table 1. Subjects in the energy restriction group are referred to as the weight-loss group (WLG) in contrast to the subjects in the weight-maintenance group (WMG). There was a similar sex distribution in both groups: 16 males and 16 females in WMG compared with 19 males and 21 females in the WLG. There were no significant differences in means of weight, height, waist, hip, waist-to-hip ratio, fasting plasma glucose and insulin concentrations, and blood pressure at baseline (TABLE 1, TABLE 2). Our approach aimed to compare the impact of dietary profile in the WLG that aligns with health guidelines and would be expected to have a positive effect on insulin sensitivity and phenotypic flexibility. We compared this to a dietary macronutrient profile commonly consumed in Europe in the WMG that would be aligned with a deterioration in insulin sensitivity and phenotypic flexibility.

**TABLE 1:** Changes in body composition and biomarkers induced by weight loss

		WLG before			WLG after			WMG before			WMG after			*P* value
		Mean	SEM	*N*	Mean	SEM	*N*	Mean	SEM	*N*	Mean	SEM	*N*	
Energy intake	kcal/d	1834.3	88	39	1343.1	80	36	1999.7	118	32	1795.7	90	31	0.005
BW	kg	84.29	2.0	40	78.66	1.92	40	83.40	2.40	32	83.46	2.35	32	<0.0001
BMI	kg/m^2^	29.31	0.47	40	27.36	0.47	40	29.03	0.44	32	29.07	0.45	32	<0.0001
Hip	cm	109.37	1.28	40	105.81	1.27	40	107.80	0.97	32	107.47	1.08	31	<0.0001
Waist	cm	100.0	1.71	40	95.07	1.80	40	99.03	1.86	32	98.24	1.92	31	0.006
Total AT	L	35.83	1.71	38	30.49	1.51	37	33.02	1.63	30	33.40	1.66	30	<0.0001
Subcutaneous AT	L	27.77	1.55	38	23.70	1.33	37	25.38	1.52	30	25.55	1.56	30	<0.0001
Internal AT	L	8.06	0.45	38	6.79	0.41	37	7.64	0.41	30	7.85	0.42	30	<0.0001
Nonabdominal internal AT	L	3.47	0.18	38	3.09	0.17	37	3.38	0.14	30	3.47	0.16	30	<0.0001
Nonabdominal subcutan. AT	L	19.53	1.06	38	16.84	0.92	37	17.96	1.01	30	18.06	1.04	30	<0.0001
Intra-abdominal AT	L	4.59	0.29	38	3.71	0.26	37	4.26	0.31	30	4.38	0.32	30	<0.0001
Abdom. subcutan. AT	L	8.24	0.52	38	6.86	0.46	37	7.42	0.54	30	7.48	0.55	30	<0.0001
Periphery AT	L	23.00	1.14	38	19.40	1.11	38	21.34	1.08	30	21.54	1.10	30	0.0035
Trunk AT	L	12.83	0.66	38	10.57	0.60	37	11.68	0.63	30	11.86	0.64	30	<0.0001
Liver fat	AU	4.35	0.79	38	2.31	0.32	33	4.81	0.94	29	5.06	1.02	30	0.0008
DBP	mmHg	79.53	1.23	40	71.85	1.57	39	75.14	1.65	32	76.06	1.71	32	0.0006
SBP	mmHg	128.10	2.08	40	121.08	1.80	39	124.92	2.02	32	126.09	2.04	30	0.0036
s-E-Selectin	ng/mL	37.48	3.64	40	29.72	2.66	39	32.06	3.93	32	31.23	3.09	31	0.0291
Leptin	ng/mL	15.97	1.36	40	9.98	1.20	39	14.63	1.46	32	14.68	1.63	31	<0.0001
Total cholesterol	mmol/L	5.40	0.17	39	4.97	0.15	39	5.16	0.17	32	5.36	0.16	32	0.0016
LDL cholesterol	mmol/L	3.12	0.16	39	2.82	0.14	39	2.89	0.16	31	3.02	0.15	32	0.0174

AT, adipose tissue; WLG, weight-lowering group; WMG, weight-maintaining group; DBP, diastolic blood pressure; SBP, systolic blood pressure. Results are presented as means ± SEM. *P* values refer to the interaction between group (WLG × WMG) and time (before × after intervention) in a mixed model analysis followed by multiple comparisons (Sídák).

**TABLE 2:** Weight loss–induced changes in plasma levels of markers of insulin sensitivity at fasting and during dietary challenges

		WLG before		WLG after		WMG before		WMG after		*P* value WLG before × after	*P*value WMG before × after	*P* value interaction arm × visit
		Mean	SEM	Mean	SEM	Mean	SEM	Mean	SEM			
Markers measured during OGTT at *t*=0 min												
Insulin	mIU/L	15.27	0.92	13.56	1.10	16.38	1.51	16.28	1.01	0.175	0.989	0.303
Glucose	mmol/L	5.20	0.09	5.02	0.08	5.13	0.10	5.02	0.09	0.018	0.275	0.483
Triglycerides	mmol/L	1.31	0.07	1.12	0.09	1.24	0.09	1.31	0.11	0.013	0.487	0.009
Ala	μmol/L	326.8	12.2	312.4	11.59	322.3	15.1	331.2	17.0	0.448	0.782	0.223
Glu	μmol/L	52.74	5.12	48.58	4.09	54.66	4.9	57.11	5.40	0.658	0.871	0.374
Leu	μmol/L	126.8	4.03	123.2	3.54	131.3	5.0	133.6	6.30	0.657	0.849	0.356
Trp	μmol/L	54.43	1.54	52.38	1.57	54.66	1.64	54.25	2.28	0.220	0.948	0.399
Tyr	μmol/L	62.28	1.91	56.87	1.93	61.98	1.61	61.38	2.47	0.008	0.948	0.077
Val	μmol/L	210.2	5.87	205.8	5.46	216.7	7.62	216.0	9.56	0.719	0.992	0.686
Propionylcarnitine	μmol/L	0.29	0.02	0.28	0.01	0.33	0.02	0.34	0.02	0.349	0.442	0.087
Ser	μmol/L	108.4	3.70	121.5	4.45	107.2	3.30	109.1	5.21	0.004	0.900	0.067
Gly	μmol/L	239.4	12.1	267.9	14.30	234.1	12.1	232.6	15.4	0.003	0.983	0.019
Markers measured during the OGTT expressed as area under the curve												
Insulin	AUC	15,062	1373	12238	1246	15,018	1669	13939	1322	0.003	0.963	0.046
Glucose	AUC	1474	55	1375	48	1489	63	1466	62	0.024	0.830	0.192
Triglycerides	AUC	306	18	271	23	306	23	309	26	0.044	0.984	0.096
Ala	AUC	77,695	2073	71,432	2506	78,956	2806	79,333	2412	0.008	0.984	0.039
Glu	AUC	8655	887	6726	626	9266	719	9502	885	0.001	0.910	0.010
Leu	AUC	23,146	988	20,758	734	23,485	784	24,682	949	0.003	0.262	0.001
Trp	AUC	11,916	273	11,227	366	11,803	336	12,070	305	0.024	0.608	0.019
Tyr	AUC	11,577	389	10,553	367	11,827	406	12,078	362	0.005	0.739	0.010
Val	AUC	43,243	1089	40,448	1360	44,359	1400	45,005	1420	0.013	0.810	0.024
Propionylcarnitine	AUC	64	3	57	3	73	5	73	4	0.048	0.999	0.126
Ser	AUC	22,100	687	23,339	913	21,968	785	22,460	755	0.002	0.576	0.115
Gly	AUC	54,242	2738	57,676	3241	52,949	2867	53,873	2783	0.032	0.803	0.234
Markers measured during the MMTT expressed as area under the curve												
Insulin	AUC	19,533	1575	16,687	1348	18,960	1414	17,693	1572	0.005	0.259	0.335
Glucose	AUC	2403	42	2316	38	2419	43	2378	50	0.102	0.654	0.506
Triglycerides	AUC	855	53	764	44	933	85	814	64	0.067	0.382	0.665
Ala	AUC	156,275	3480	150,327	4296	160,771	5695	157,140	5694	0.565	0.518	0.889
Glu	AUC	19,606	1172	16,422	1098	21,014	1734	20,524	1702	0.004	0.747	0.107
Leu	AUC	67,793	1683	65,719	1615	70,621	2049	71,130	2587	0.399	0.954	0.314
Trp	AUC	24,033	537	23,213	587	23,587	535	23,704	714	0.202	0.984	0.234
Tyr	AUC	30,425	709	28,943	843	30,528	918	30,677	1036	0.096	0.999	0.181
Val	AUC	101,730	2250	99,049	2339	103,753	3066	103,553	3642	0.252	0.801	0.582
Propionylcarnitine	AUC	138	6	129	6	156	10	150	9	0.160	0.707	0.550
Ser	AUC	51,436	1845	56,298	2020	51,718	2311	49,508	2410	0.003	0.388	0.003
Gly	AUC	111,582	5468	122,677	6082	110,783	5792	107,325	5039	0.002	0.553	0.003

MMTT, mixed-meal tolerance test; WLG, weight-lowering group (*N =* 40); WMG, weight-maintaining group (*N =* 32); Ala, alanine; Glu, glutamine; Leu, leucine; Trp, tryptophan; Tyr, tyrosine; Val, valine; Ser, serine; Gly, glycine. Results are presented as means ± SEM. Metabolites in highlighted lines are positively correlated to insulin sensitivity. *P* values refer to multiple comparisons (Sídák) between WLG before and after the intervention, WMG before and after the intervention, and for the interaction arm (group) × visit (before and after intervention) that followed a mixed model analysis.

The dietary macronutrient content in the WLG reflected nutritional recommendations in the United Kingdom. Approximately 50% of energy derived is from carbohydrates, of which more than 18 g of dietary fiber, 35% from fat, and 15% from protein. Energy intake was 20% less than the estimated energy expenditure [7,8]. The diet was based on 5 main food groups (grains, fruit and vegetables, meat and fish, dairy, and fats), with a dietary profile aligned to Dietary Approaches to Stop Hypertension (DASH) recommendations [9]. This diet has been demonstrated to improve insulin sensitivity and cardiovascular disease risk factors [10]. The WMG followed a diet matching their usual energy expenditure. The diet was based on the average intake in the EU and included approximately 45% energy derived from carbohydrates, 40% from fat, and 15% from protein [10].

Both groups were contacted individually at weeks 0, 4, 8, and 12, and with telephonic interviews on weeks 2, 6, and 10. The aim was to encourage dietary compliance and reduce variability in weight loss. Diets were individually composed to comply with the personal dietary habits of everyone. Both groups completed 7-d food diaries during weeks 1–13 to monitor dietary changes. Both groups were instructed to keep exercise at habitual levels. More details about the intervention study can be found in the Supplementary Methods.

### Randomization

Randomization was carried out using an online system for clinical trials called Sealed Envelope (https://www.sealedenvelope.com/). Stratified randomization based on sex, age, and BMI was used to allocate volunteers to each group.

### Dietary compliance

Dietary compliance was monitored by estimating the change in fat-free and fat mass over time [11]. We also estimated change in diet quality using urinary metabolomic dietary model [12]. The urine samples were prepared with a pH 7.4 phosphate buffer for ^1^H-NMR spectroscopy as previously described [13] and were analyzed at 300 K on a 600 MHz spectrometer (Bruker BioSpin) using a standard 1-dimensional pulse sequence with water presaturation [13]. The urinary metabolic profiles were projected into a previously validated urinary metabolomic dietary model [12].

### Challenge tests

A major aspect of phenotypic flexibility is the ability to recover from transient metabolic perturbations reflected in changes in plasma levels of metabolites and signaling molecules when challenged with food intake. Two dietary challenges were carried out after overnight fasting at baseline and after 12 wk of the intervention to assess changes in metabolism:

1. OGTT, when blood samples were taken at *t*=0, 15, 30, 60, 120, and 240 min after the intake of 75 g glucose in 200 mL filtered water. This allows a standardized method to assess glucose homeostasis.
2. MMTT, based on a liquid, high-fat, and high-glucose drink [4,5]. Blood samples were taken at *t*=0, 30, 60, 120, 240, 360, and 480 min after intake of a 200 mL test meal containing 75 g glucose, 60 g palm oil, and 20 g casein. This allows for the assessment of a standard dose of carbohydrates, fat, and protein on metabolism.

Further details about the dietary challenges are presented in Supplementary Methods.

### Measurement of biomarkers

To assess changes in metabolism, multiple biomarkers including glucose, insulin, HbA1c, TG, LDL-cholesterol, total cholesterol (tChol), GGT, creatinine, uric acid, nonesterified fatty acids, glucagon, intercellular adhesion molecule 1 (ICAM1), vascular cell adhesion protein (VCAM), s-E-selectin, GLP-1, peptide YY (PYY), IL 1 beta (IL-1β), IL-6, IL-8, IL-10, IL-18, and IL-1 receptor antagonist (IL-1RA) were quantified in plasma using antibody-based and enzymatic methods, following manufacturer’s instructions. Insulin sensitivity indices such as HOMA-IR and HOMA for β-cell function (HOMA-β), and Matsuda index (MI) were calculated [[14], [15], [16], [17], [18]]. Detailed information about these measurements is presented in Supplementary Methods.

### Targeted and untargeted metabolomics platforms

Acylcarnitines (24) amino acids (22), biogenic amines (12), glycerophospholipids (90), and sphingolipids (15) were quantified using the LC with tandem MS (LC-MS/MS)-based AbsoluteIDQ p180 Kit (Biocrates Life Sciences AG), following the manufacturer’s protocol. Additional acylcarnitines (26) were quantified after extraction with methanol in the presence of deuterated standards and butylated before analysis using liquid chromatography coupled to a Sciex 5500 mass spectrometer (Sciex) (LC-MS/MS) following a previously described method [19]. The 13 most abundant bile acids (BA) in plasma were quantified using an adapted method [20]. Briefly, 10 μL plasma was mixed with deuterated internal standards. After deproteinization with methanol, samples were evaporated to dryness, reconstituted in methanol:water (1:1), and injected into the LC-MS/MS system [21].

For the GC-MS analysis, metabolites were extracted from 40 μL plasma by ice-cold methanol:H_2_O (8:1) in a sample:solvent ratio of 1:10. After centrifugation (13,200 × *g*, 4 min, 4°C), 200 μL of supernatant was dried under vacuum. A 2-step derivatization was performed using an autosampler (Agilent 7693, Agilent Technologies) by incubating the samples with methoxyamine hydrochloride (20 mg/mL in pyridine) for 30 min at 45°C, followed by the addition of *N*-methyl-*N*-trimethylsilyl-triflouroacetamide, and a second incubation for 30 min at 45°C. Immediately after derivatization, each sample was submitted to GC-MS analysis (Agilent 6890N GC coupled to an Agilent 5975C inert XL; Agilent Technologies). The gas chromatograph was equipped with a 30-m DB-35MS capillary column (Agilent J&W GC Column). Metabolites were eluted by a temperature gradient from 80°C and rising by 11°C/minute to 325°C with 5 min hold at 325°C. Metabolite identification and quantification were accomplished using the Metabolite Detector software. Metabolites were identified according to their retention time and spectra similarity against the Golm metabolome database.

Plasma samples were randomized to exclude batch variation, ensuring a proportional number of samples from the 2 dietary challenges, collected before and after the weight loss intervention and from men and women. Quality control plasma samples (Recipe chemicals and instruments) were included in each batch of samples to control for analytical drifting.

### Magnetic resonance imaging for determination of body composition

Detailed methodology of MRI measurements has been reported elsewhere [[22], [23], [24]] to quantify total and regional adipose tissue (AT) depots, as well as the fat content of the liver, pancreas, and soleus and tibialis muscles. A typical example of these measures from a single volunteer is shown in Supplemental Figure 2.

### Sample size estimation

Our primary outcome was insulin sensitivity. Data for the power analysis were taken from Blumenthal et al. [10]. This was the only trial that reported the impact of weight loss and change in dietary quality on insulin sensitivity when the protocol was developed. In this trial, weight loss and improved dietary quality were associated with improved insulin sensitivity assessed by OGTT and lower total cholesterol. The difference between the means in the Blumenthal et al. [10] study was 0.18 with a standard deviation of 0.2 (a decrease that was also associated with significant improvement in several plasma risk factors such as total cholesterol and triglycerides), assuming an alpha of 5% and power of 90 and 2-tailed test to detect a change in insulin sensitivity. Thus, we needed 37 participants per group. Allowing for a dropout of 30%, we aimed to recruit 50 volunteers per group.

### Data integration and statistical analyses

All NutriTech data were collected in a distributed database specifically designed to handle multi-omics human nutritional intervention studies performed at multiple sites, the “nutritional phenotype database” (dbNP http://www.dbnp.org/). Data were checked for normality using Shapiro–Wilks and all results are presented as means ± SEM. In case of missing data, the imputation was done by averaging the nearest neighbors. Because most of the missing data were derived from time series analyses (the dietary challenges), this was the method of choice. A mixed model analysis of variance was applied to fasting data including intervention (energy restriction/weight maintenance) and term (baseline/follow-up) as fixed factors and their interaction. The subject was used as a random factor. Detailed statistical procedures are described in the Supplementary Material. Pearson correlation coefficients were calculated and reported together with *P* values as well as the number of samples used in the calculation. Partial least squares discriminant analysis (PLS-DA) was used to build a model to identify discriminating metabolites in a subset of study subjects. Monte Carlo cross-validation was used to train 1000 models and obtain an aggregate predicted score for each sample when part of the test set with one-fifth of the data held out at random in each iteration.

## Results

### Response to intervention

#### Weight loss.

The 12 wk energy restriction promoted a significant reduction in body weight (BW) in the WLG displaying a mean reduction of 5.6 kg (*P <* 0.0001), whereas the WMG did not display a reduction in BW (Table 1). Despite meeting the target weight loss, there was a wide individual variation in the response to the intervention, with weight loss in WLG ranging from 0.1 to 17.5 kg (Supplemental Figure 3).

#### Impact of the intervention of dietary intake.

We observed a significant reduction in self-reported energy intake in the WLG group (average energy intake reduction 503.9±85.3 kcal/d, *P <* 0.0001) as compared with a nonsignificant alteration in the WMG (average energy intake reduction 171.6±98.3 kcal/d, *P =* 0.08; Table 1). Participants in the WLG also profited from improved dietary quality assessed by DASH score (2.2±0.21 compared with 3.6±0.22, *P <* 0.001), which was not seen in the WMG (1.9±0.2 compared with 1.8±0.19, *P <* 0.68). Waist and hip circumferences were reduced in the WLG, although the waist:hip ratio was unaffected. Systolic and diastolic blood pressure decreased upon weight loss in the WLG (5.5% and 8.4% decreases, *P* = 0.005 and 0.0007, respectively), whereas no effect was observed in the WMG (Table 1).

#### Dietary compliance.

**FIGURE 1:**
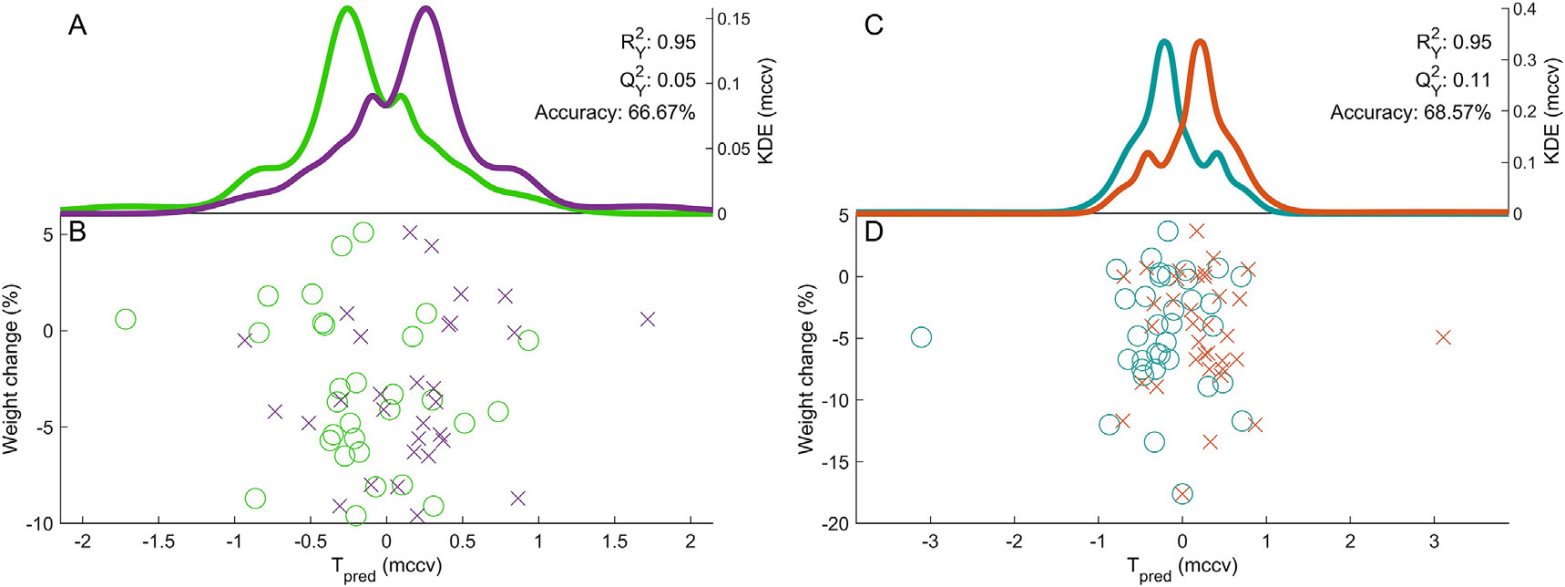
Dietary predictions using urinary metabolomics in the repeated measures design. (A, B) weight-maintaining group (WMG) diet week 1 is green; week 12 is purple. (C, D) weight-lowering group (WLG) diet – week 1 is cyan; week 12 is orange. (A, C) Kernel density estimates (KDEs) of the Monte Carlo Cross Validation (MCCV) Repeated Measures Partial Least Squares (RM-PLS) predictions. (B, D) Predicted scores (*T*_pred_) against the weight change (in %). Using a one-sample t-test to assess the difference between weeks 1 and 12, there was no significant difference in the weight-maintenance group at *P =* 0.06 but there was in the weight loss group toward the healthy eating profile at *P =* 0.03.

There was a strong relationship between percentage weight loss and estimated daily dietary energy reduction based on actual weight loss (*r =* 0.83, *P <* 0.001). In the WLG 10/36 volunteers had estimated daily energy reduction based on actual weight loss <75% of the prescribed diet. The heterogeneity of the weight loss appeared to be due to dietary compliance. Urine metabolomics assessment of diet quality showed a slight improvement in the diet quality for WLG but not in the WMG (Figure 1).

### Markers of insulin sensitivity and phenotypic flexibility during dietary challenges

There was no significant difference in fasting glucose, insulin, HOMA IR, or HOMA %β during the 12 wk intervention between the WLG and the WMG in the OGTT (Table 3). However, there was a significant decrease in fasting insulin and HOMA IR in the MMTT. The estimation of insulin sensitivity using the MI suggests an increase in insulin sensitivity after weight loss in the OGTT but not in the MMTT.

**TABLE 3:** Fasting and mean postprandial values for glucose, insulin, HOMA-IR, Β-cell function, and the Matsuda Index fasting and postprandial parameters from OGTT and MMTT tests

Variable	WLG					WMG					ANCOVA
	Week 1		Week 12			Week 1		Week 12			
	Mean	SEM	Mean	SEM	Δ Change	Mean	SEM	Mean	SEM	Δ Change	*P* value
OGTT											
Fasting glucose, mmol/L	5.1	0.1	5.0	0.1	-0.17 (-0.3,-0.04)	5.1	0.1	5.0	0.1	-0.1 (-0.3,0.04)	0.610
Fasting insulin, μU/mL	15.2	0.9	13.6	1.1	-1.6 (-3.5,0.4)	16.4	1.5	16.3	1.0	-0.15 (-2.2,1.9)	0.240
HOMA IR	1.7	0.1	1.5	0.1	0.19 (-0.4,0.02)	1.8	0.2	1.8	0.1	0.02 (-0.3,0.2)	0.090
HOMA %β	127.5	5.8	125.5	7.3	-1.94 (-12.8,8.9)	134.0	5.4	141.8	6.7	7.8 (-3.4,19.4)	0.170
Matsuda Index	4.0	0.3	4.8	0.3	0.8 (0.3,1.3)	3.9	0.3	4.0	0.3	0.15 (-0.2,0.6)	0.030
MMTT											
Fasting glucose, mmol/L	5.1	0.1	5.0	0.1	-0.17 (-0.3,-0.04)	5.0	0.1	5.0	0.1	0.02 (-0.9,1.0)	0.100
Fasting insulin, μU/mL	18.5	1.3	14.8	0.1	-3.6 (-27.7,8.8)	18.0	1.2	19.1	1.9	1.1 (-13.2,23.9)	0.003
HOMA IR	2.5	0.1	1.9	0.1	-0.5 (-3.2, 1.1)	2.4	0.1	2.5	0.1	0.13 (-1.7,3.0)	0.003
HOMA %β	167.2	2.5	153.5	1.8	-13 (-144,88)	170.0	2.1	176.0	3.3	4.6 (-118,161)	0.053
Matsuda Index	4.4	0.6	5.0	0.4	0.5 (-13,4.5)	3.9	0.3	4.4	0.4	0.5 (-3.4,8.7)	0.510

MMTT, mixed-meal tolerance test; WLG, weight-lowering group; WMG, weight-maintaining group. Values expressed mean and standard error and delta change from baseline. Between-subject effects reported in tables were assessed using ANCOVA with post values as dependent variables, type of intervention as a fixed factor (0 = weight loss, 1 = maintenance), and baseline data as a covariate.

**FIGURE 2:**
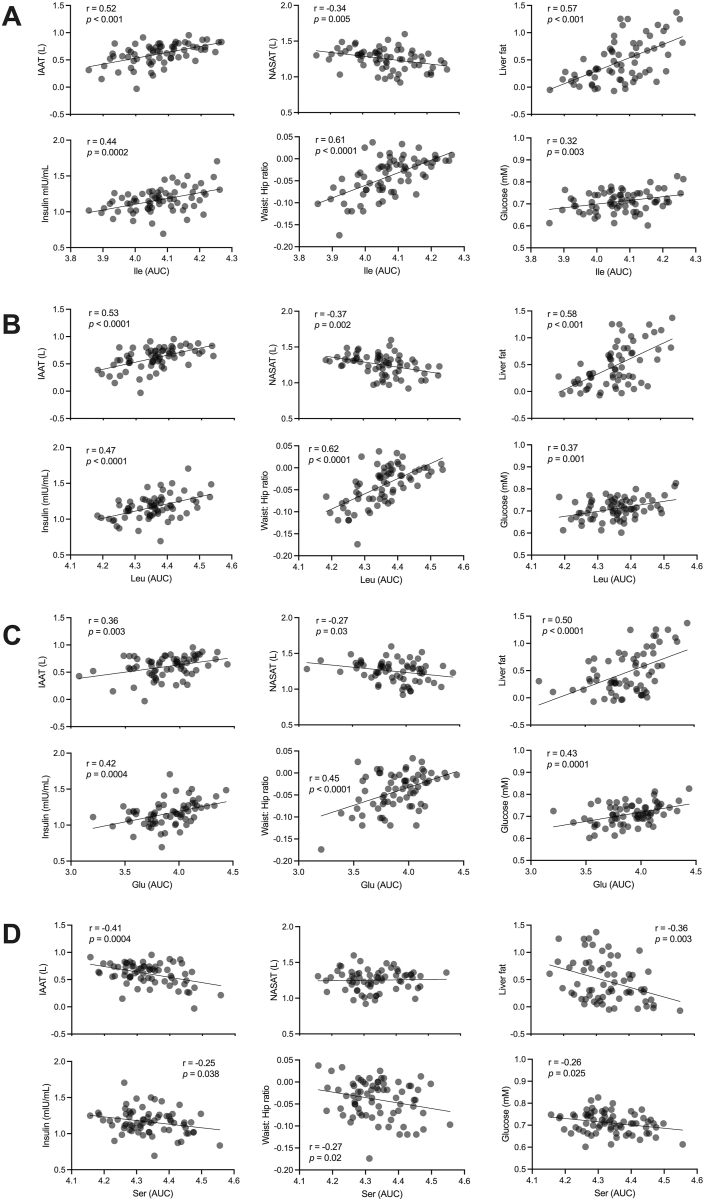
Associations between markers of insulin sensitivity with adipose tissue and plasma glucose and insulin levels before the weight-loss intervention. (A) Pearson correlations of different markers with isoleucine. (B) Pearson correlations of different markers with leucine. (C) Pearson correlations of different markers with glutamate. (D) Pearson correlations of different markers with serine. The area under the curve data were derived from OGTT. Data were Log-transformed. Abbreviations: IAAT, intra-abdominal adipose tissue; NASAT, nonabdominal subcutaneous adipose tissue. *N =* 72.

In our present study, we confirm previous observations on the relationship between body composition and markers of insulin sensitivity, although the study population primarily included normoglycemic individuals according to WHO definitions [25]. Established metabolomics markers of obesity and insulin sensitivity showed strong correlations with the volume of specific fat depots, waist:hip ratio, and glucose and insulin postprandial curves. For instance, the AUC the OGTT (OGTT AUC) of isoleucine, leucine, and glutamate was positively correlated with intra-abdominal AT, waist:hip ratio, and liver fat content. They were also positively correlated with the OGTT AUC of glucose and insulin (Figure 2). However, the OGTT AUC of these amino acids displayed a negative correlation with nonabdominal subcutaneous AT, confirming previous reports that deposition of subcutaneous fat might promote a smaller metabolic burden than the intra-abdominal deposition of AT [26,27]. The OGTT AUC of serine, an amino acid reported in association with insulin sensitivity, displayed negative correlations with intra-abdominal AT, waist:hip ratio, and hepatic fat content. The plasma concentration of serine during the OGTT was also negatively correlated with the OGTT AUC of glucose and insulin (Figure 2D). When using the same correlations for fasting data rather than OGTT AUC, similar observations were made (data not shown).

### Dietary challenges as a tool to assess weight loss–induced metabolic improvements

**FIGURE 3:**
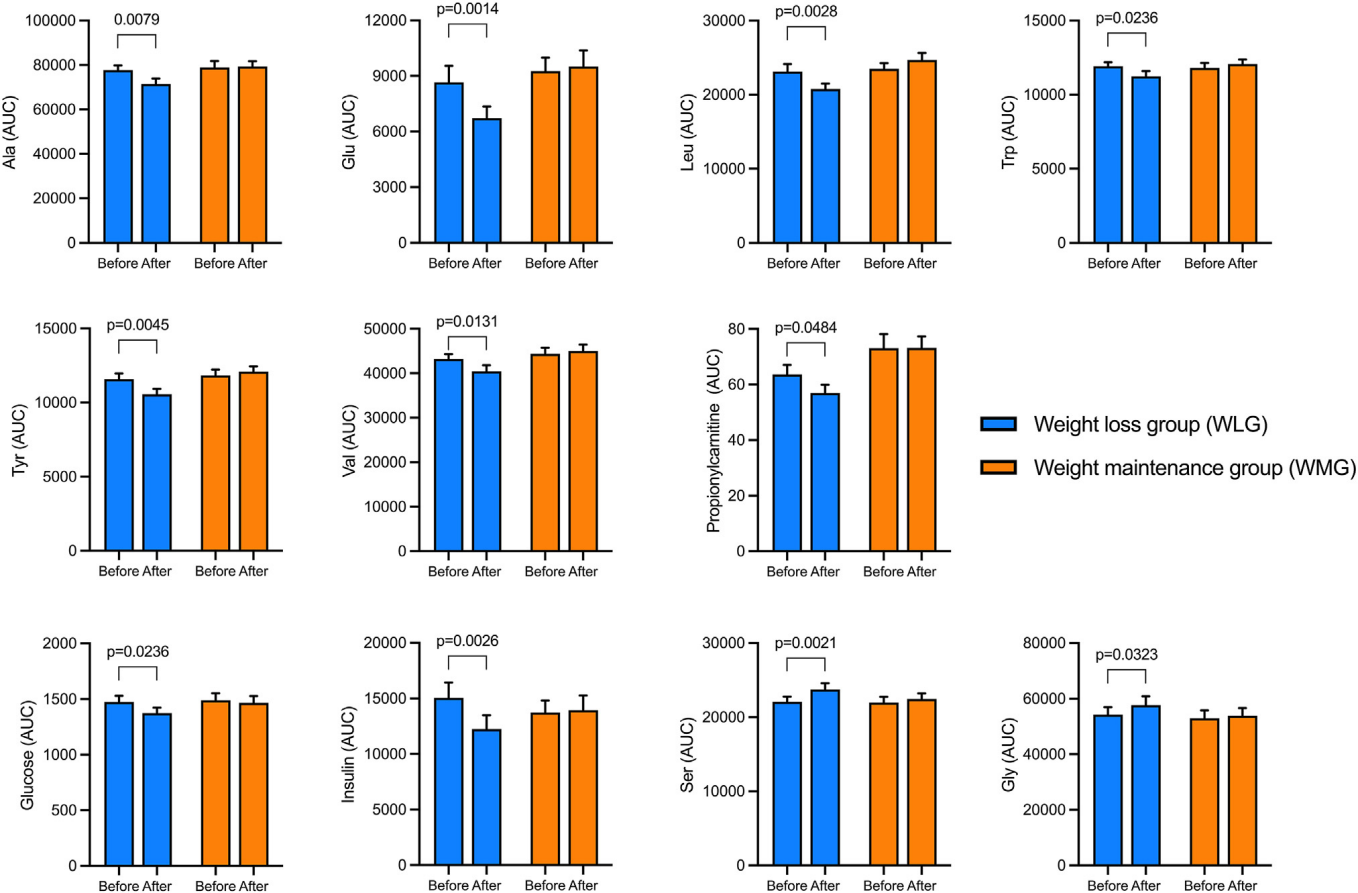
Weight loss–induced changes in plasma concentration of markers of insulin sensitivity during an OGTT. Data are presented as means ± SEM. The adjusted *P* value is given for statistically significant differences after a mixed model analysis followed by multiple comparisons (Sídák); Weight-lowering group (WLG), *N* = 40. Weight-maintaining group (WMG), *N* = 32.

Weight loss failed to induce changes in fasting plasma concentrations of insulin, valine, and leucine, as well as other amino acid-based markers of insulin sensitivity (Table 2). However, during the OGTT, individuals from the WLG displayed lower plasma concentrations of these markers after the weight loss. The OGTT AUC of alanine, glutamate, leucine, tryptophan, tyrosine, and valine – markers of insulin resistance reported in previous studies [28,29] – were reduced after weight loss in the WLG, whereas no changes were observed in the WMG (Table 2 and Figure 3). On the contrary, the AUC of serine and glycine – metabolites previously associated with insulin sensitivity – were increased during the OGTT in the WLG after the dietary intervention by ∼5% (*P <* 0.04). The AUC of insulin during OGTT responded to weight loss with a 19% reduction (*P =* 0.007) in contrast to fasting insulin, which remained unchanged (Table 2 and Figure 3). During the MMTT, these effects were mostly unnoticed. Only insulin and glutamate exhibited reduced plasma concentrations during MMTT after weight loss, whereas serine and glycine had increased AUC during this dietary challenge (Table 2 and Figure 3). These results suggest that a dietary challenge can help detect subtle metabolic changes after weight loss, and OGTT seems to perform better than MMTT in this regard.

### Weight loss intervention improves body composition and alters metabolic biomarkers

**FIGURE 4:**
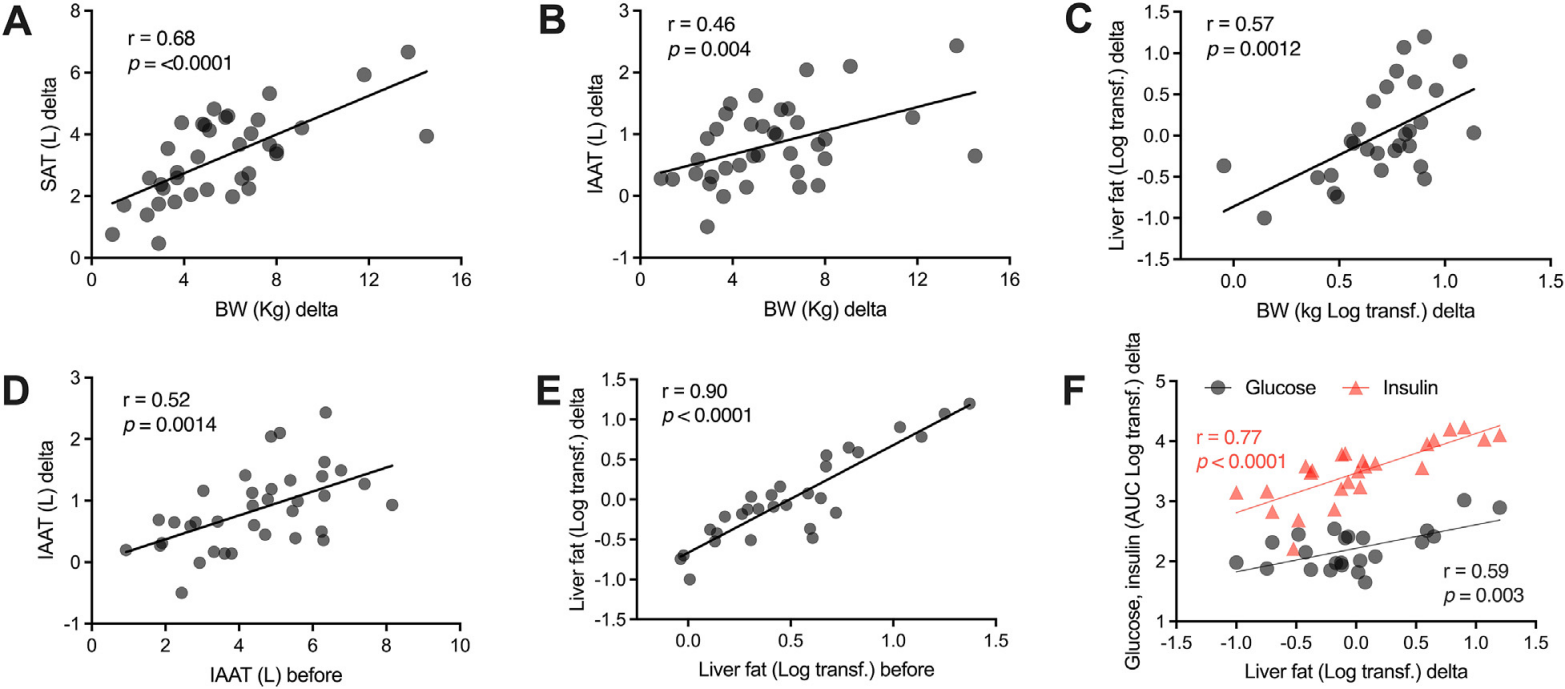
Associations between weight loss–induced changes in adipose tissues, ectopic fat, and markers of glucose metabolism. Two-tailed Pearson correlation analyses. When indicated, an area under the curve was derived from the OGTT. Some variables were log-transformed to facilitate visualization of the associations. Delta was calculated by subtracting the value after weight loss intervention from the value before intervention. Abbreviations: IAAT, intra-abdominal adipose tissue; BW, body weight; SAT, subcutaneous adipose tissue. Only individuals from weight-lowering group (WLG) are included (*n*≈40).

Following the weight loss intervention there were significant reductions in total and regional AT depots in the WLG, without effects in the WMG (interaction between group and time, *P <* 0.0001; Table 1). The mean total AT loss following the intervention was 5.3 L. The magnitude of AT reduction was greatest for the intra-abdominal AT (19.2%), and the smallest reductions were observed in the nonabdominal internal AT (10.9%), suggesting a preferential reduction of abdominal fat. The weight loss correlated positively with the reduction in the volume of subcutaneous AT (*r =* 0.68, *P <* 0.0001), intra-abdominal AT (*r =* 0.46, *P =* 0.004), and liver fat (*r =* 0.57, *P =* 0.0012) (Figure 4A–C). Although large interindividual differences were observed, there was a reduction in liver fat content in the WLG group (46.8%, *P =* 0.0008, Table 1).

The amount of intra-abdominal AT at baseline correlated with the reduction in this AT depot following weight loss (*r =* 0.52, *P =* 0.0014; Figure 4D); no such relationship was observed for total AT. The baseline liver fat content was even more strongly correlated with the reduction in liver fat (*r =* 0.90, *P <* 0.0001, Figure 4E). The reduction in liver fat content was also correlated with a reduction in the AUC of glucose (*r =* 0.59, *P =* 0.0026) and insulin (*r =* 0.77, *P <* 00001) during the OGTT (Figure 4F).

In parallel to changes in body composition, fasting plasma concentrations of glucose, TG, TC, and LDL cholesterol presented small but significant reductions (*P <* 0.03) in the WLG upon the 12 wk of energy restriction (TABLE 1, TABLE 2). Blood pressure, leptin, and s-E-selectin also had their plasma concentrations reduced after weight loss in the WLG (Table 1).

### Metabolic effects of weight loss related to insulin sensitivity and dietary quality

The effect of energy restriction on metabolic parameters and phenotypic flexibility was surprisingly small, despite the homogeneous study population and the successful weight loss. This may be due to the large difference in responses to weight loss among subjects in WLG, with weight loss ranging from 1.1% to 13.4%. In addition to this wide spectrum of responses to the weight loss intervention, some volunteers of the WLG experienced improved insulin sensitivity assessed with the MI, whereas others had no change in insulin sensitivity, despite considerable weight loss.

We investigated this phenomenon using information derived from the metabolomics analysis. To ensure that all subjects complied with the energy restriction, only volunteers who had a minimum weight loss of 5% were selected (26/40 volunteers). In the volunteers, variations in the MI after weight loss ranged from −15% to 274% and served to rank the participants into 2 groups: one including subjects with an increase of ≥35% in MI, named improvement group (*n =* 13), and a second group with <35% of increase or even a decrease in MI – the no-improvement group (*n =* 13). These 2 groups had similar changes in BW and reduction in different adipose depots in response to the intervention, losing 16%–20% of total adipose mass, but had very different changes in insulin sensitivity as measured by the MI (Table 4).

**TABLE 4:** Weight loss–induced changes in anthropometric data, clinical chemistry, and the Matsuda Index in participants with or without improved insulin sensitivity during the OGTT

	Improvement				No improvement				*P* values		
	Βefore interv.		Αfter interv.		Βefore interv.		Αfter interv.		Interaction	Improvement (before ×after)	No improvement (before × after)
	Αverage	SEM	Αverage	SEM	Αverage	SEM	Αverage	SEM			
Matsuda Index (0–120 min)	2.86	0.35	4.90	0.50	3.91	0.65	3.97	0.57	<0.0001	<0.0001	0.971
BMI (kg/m^2^)	29.14	0.86	26.55	0.88	29.83	0.92	27.56	0.90	0.351	<0.0001	<0.0001
Body weight (kg)	83.16	3.15	75.68	2.90	86.59	3.40	79.96	3.20	0.428	<0.0001	<0.0001
Total adipose tissue (L)	31.91	2.99	26.59	2.91	38.55	3.47	30.46	2.40	0.872	<0.0001	<0.0001
Subcutaneous adipose tissue (L)	24.20	2.79	20.37	2.64	30.45	3.17	23.95	2.10	0.640	<0.0001	<0.0001
Intra-abdominal adipose tissue (L)	4.62	0.46	3.53	0.42	4.57	0.38	3.45	0.28	0.670	<0.0001	<0.0001
Liver fat (arbitrary units)	6.52	1.92	2.81	0.70	3.79	1.35	1.78	0.55	0.397	0.012	0.248
Diastolic blood pressure (mmHg)	83.23	2.21	74.85	2.94	75.46	2.06	70.50	2.81	0.441	0.024	0.246
Systolic blood pressure (mmHg)	131.92	3.56	126.31	2.78	125.31	2.93	119.33	3.09	0.960	0.259	0.248
Triglycerides (mM)	1.39	0.15	1.21	0.13	1.32	0.17	1.05	0.08	0.605	0.282	0.071
LDL-cholesterol (mM)	2.92	0.27	2.53	0.23	2.67	0.24	2.78	0.19	0.066	0.088	0.793
Glucose (mM)	5.46	0.14	5.24	0.13	5.23	0.10	5.03	0.09	0.833	0.064	0.118
Total cholesterol (mM)	5.17	0.31	4.70	0.24	4.98	0.20	4.90	0.19	0.171	0.048	0.904
GGT (U/L)	25.08	6.16	16.54	3.19	33.15	11.65	17.81	2.44	0.577	0.546	0.161
	Average		SEM		Average		SEM		*P* values t-test		
Matsuda index delta (absolute)	2.04		0.30		0.05		0.17		<0.0001		
Matsuda index delta (%)	81.23		17.57		5.37		4.51		0.0003		
Body weight delta (kg)	-7.48		0.82		-6.63		0.66		0.427		
	*N*				*N*				*χ*^*2*^*P* value		
Male	8				5				0.239		
Female	5				8						

OGTT, oral glucose tolerance test; MMTT, mixed-meal tolerance test. Data are presented as means ± SEM. *N* = 13 in each group. *P* values refer to the interaction between the terms “group” and “time” after a mixed model analysis. The *P* values from the multiple comparisons (Sídák) “before × after” for the Improvement group. A t-test was performed between the average values for the changes in Matsuda index and body weight. A Chi-square analysis indicates that there is no significant difference between the proportion of males and females in each group.

**FIGURE 5:**
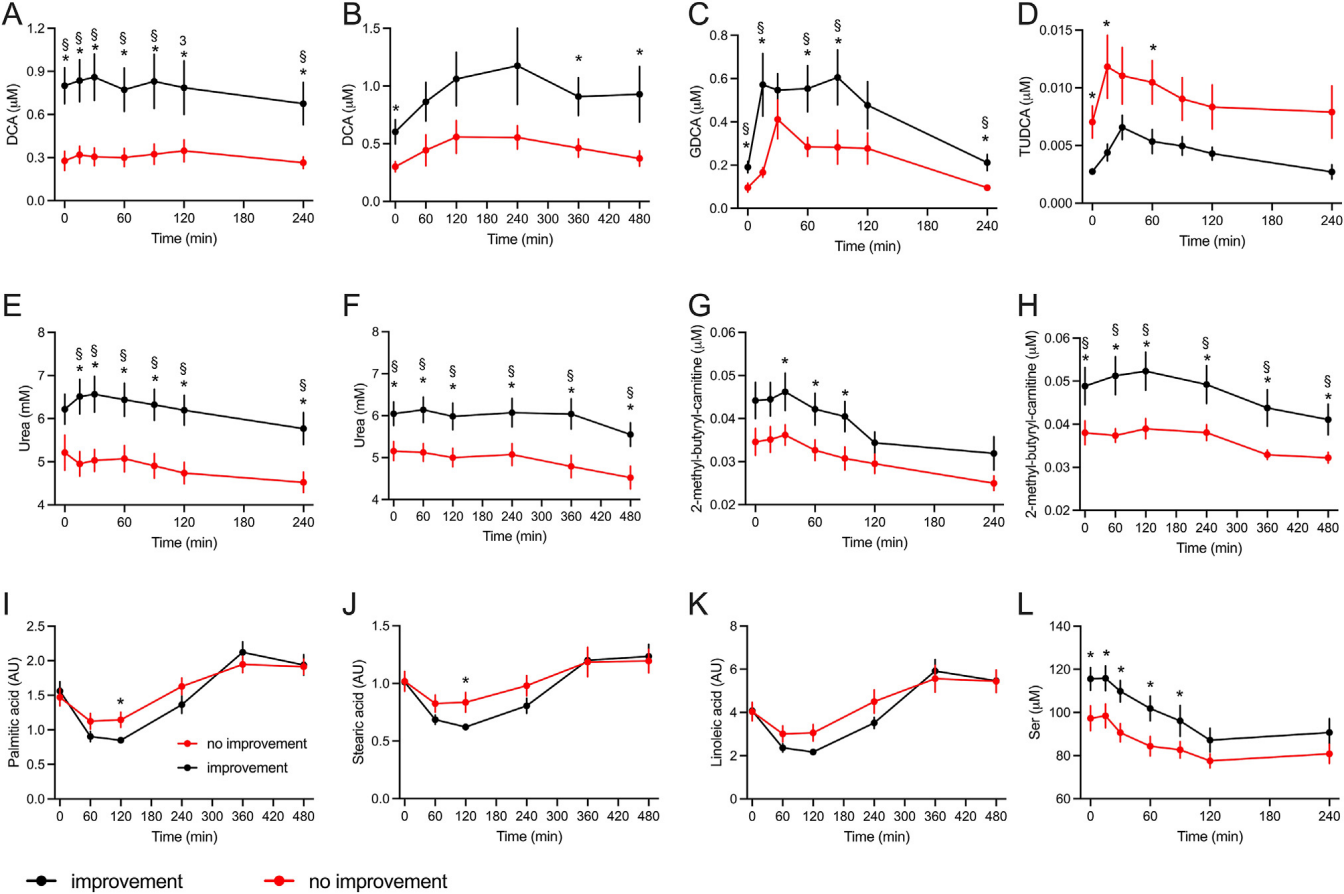
Metabolites discriminating participants with or without improved insulin sensitivity after weight loss. Data derived from the OGTT (A, C, D, E, G, and L) or mixed-meal tolerance test (MMTT) (B, F, H–K) carried out before the weight-loss intervention. DCA, deoxycholic acid; GDCA, glycodeoxycholic acid; TUDCA, tauroursodeoxycholic acid. Data are presented as means ± SEM. Differences between the 2 groups were analyzed by multiple, unpaired t-tests. False discovery rate approach by the 2-stage step-up method of Benjamin, Krieger, and Yekutieli. ∗ = *P <* 0.05. § = *q*-value < 0.05. *n* = 13 in each group.

A PLS-DA model was built on results from the metabolomics analyses of plasma sampled during the MMTT and OGTT and body composition data to identify metabolic differences between the improvement and no-improvement groups. The generated model had 3 components, *R*^2^ = 0.97 and *Q*^2^ = 0.29, and did not survive cross-validation, indicating that globally there were no differences between the plasma metabolomes of these 2 groups. This outcome is probably influenced by the small number of samples in each group (*n =* 13). Nevertheless, the model allows the identification of metabolites at different time points during the OGTT and MMTT and body composition parameters most important for the discrimination between the 2 groups (variables with higher VIP values) (Supplemental Table 2). Urea, BA, amino acids, acylcarnitines derived from the degradation of branched-chain amino acids (BCAA), as well as different glycerophospholipids and sphingomyelins, were the most discriminating metabolites for individuals who did or did not show improved insulin sensitivity after weight loss and included glycocholate (GCA), deoxycholate (DCA), tauroursodeoxycholate (TUDCA), palmitate, stearate, linoleate, urea, 2-methyl-butyryl-carnitine, and serine (Figure 5).

**FIGURE 6:**
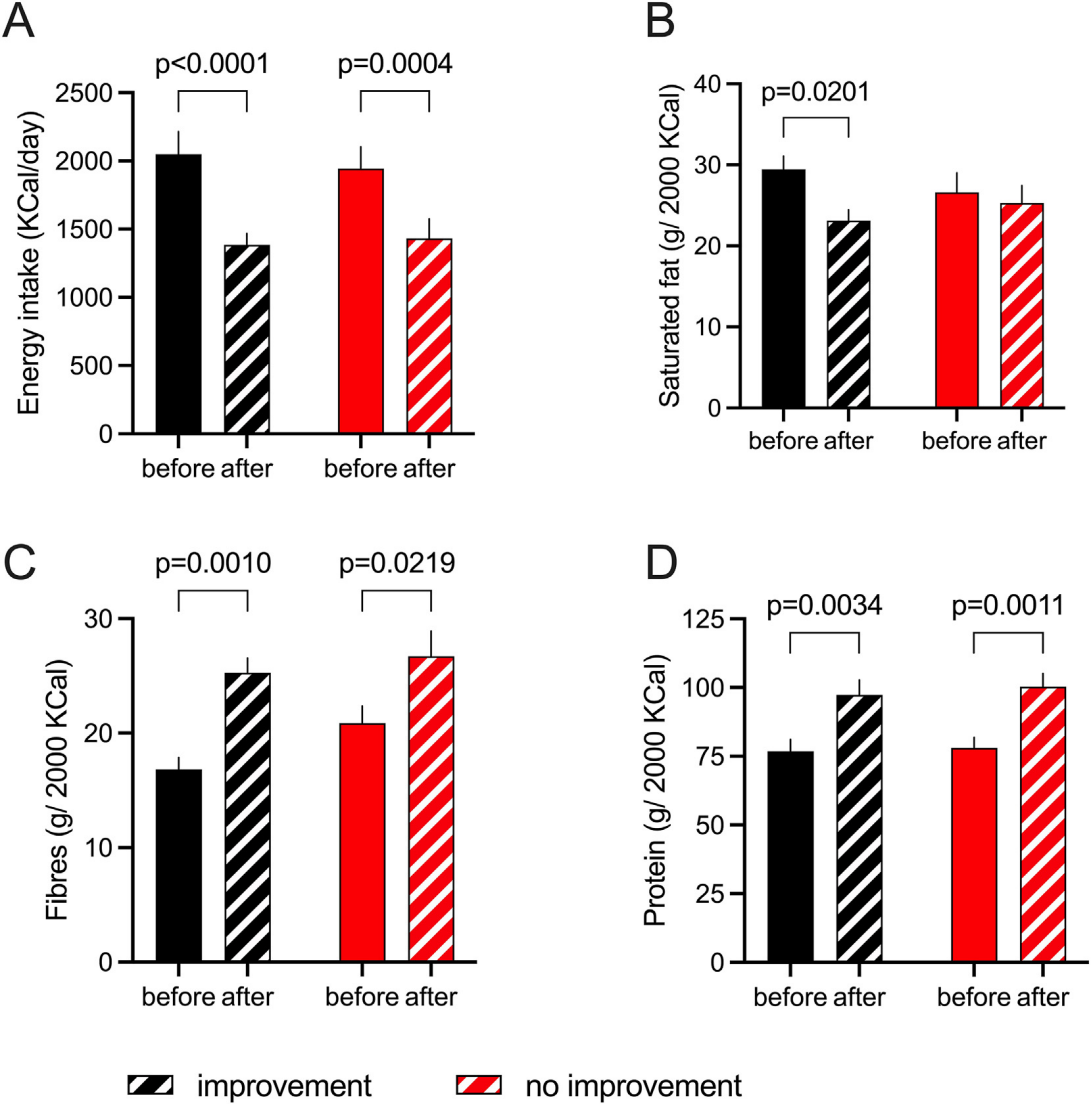
Dietary changes induced by the weight loss intervention. (A) Energy intake. (B) Intake of saturated fat. (C) Intake of fiber. (D) Protein intake. The *P* value of the Student’s t-test is indicated when significant. Data are presented as means ± SEM. The adjusted *P* value is given for statistically significant differences after a mixed model analysis followed by multiple comparisons (Sídák). *n* = 13 in each group.

The food intake records indicate that when adjusted for total energy intake, volunteers with improved insulin sensitivity had a 20% lower intake of fiber compared with the no-improvement group before the weight loss intervention*,* reaching the same level of intake after the intervention, which increased in both groups (*P =* 0.001 in improvement compared with *P =* 0.0219 in no-improvement) indicating a higher chance of fiber intake in the group with improved insulin sensitivity. Moreover, only the improvement group had a 22% reduced intake of saturated fat during the intervention (*P =* 0.02), indicating additional improvement in diet quality (Figure 6).

## Discussion

In the NutriTech study, whole-body MRI and plasma biomarker analyses were used to assess the effects of energy restriction on insulin sensitivity and metabolic health of overweight subjects. The use of dietary challenges aimed to assess whether changes in phenotypic flexibility could be more effectively detected by measuring postprandial markers rather than fasting samples. The outcome of this study indicates that despite an average BW loss of 6.4% in the WLG, only very few classical metabolic effects were observed. Although an estimated 60% of the cohort adhered to 75% of the dietary prescription, there were significant interindividual differences in visceral fat reduction induced by the weight loss intervention, which matches differences in response to the 2 dietary challenge tests. Another important finding was the variation in parameters that report insulin sensitivity. Although fasting insulin significantly decreased and HOMA-IR significantly increased after the intervention in the WLG during the MMTT, this was not observed during the OGTT. Moreover, the change in the postprandial estimation of insulin sensitivity by the MI following weight loss was also different between the OGTT and MMTT. This variation in response has been previously commented on by others [30]. We believe that some of the variability may be due to the individual phenotype response outlined below.

The dietary intervention resulted in an average weight loss of over 5 kg, accompanied by significant reductions in adipose mass, as well as waist and hip circumference, and ectopic fat mass. Consistent with previous studies on weight loss [[31], [32], [33]] and exercise interventions [34], the greatest reduction in AT mass was observed in visceral AT, which was associated with reductions in liver fat content. The baseline liver fat content for our overall cohort was low (mean 4.42 ± 6.64%). Although only a quarter of the subjects had liver fat greater than the level suggestive of nonalcoholic fatty liver disease, the WLG exhibited a substantial percentage change in liver fat (46%). This is comparable to results seen in other studies of weight loss through lifestyle modifications [31,32,[35], [36], [37], [38]], indicating that liver fat is responsive to negative energy balance [39]. However, the mean weight loss masks a wide variability in individual weight loss, which might have affected the group comparison.

The effects of weight loss on muscle lipid content are still debated [40], as some studies have reported a reduction in intramuscular cellular lipid (IMCL) following weight loss [31,32,41], whereas others have shown no changes [36,42].

The reduction in fasting plasma glucose was modest (−3,5% *P =* 0.02), and the change in fasting plasma insulin (−11%) was not statistically significant (Table 2). This may be because, despite their overweight/obese phenotype (mean BMI = 29.2 kg/m^2^), our middle-aged volunteers were normoglycemic according to the WHO criteria with only 3 individuals having fasting plasma glucose > 6.1 mmol/L [25].

The dietary challenges (OGTT and MMTT) performed before and after 12 wk of energy restriction revealed subtle changes in phenotypic flexibility when postprandial changes in plasma levels of glucose, insulin, and amino acids markers of insulin resistance (Ile, Leu, Val, Trp, Glu, and Ala) were considered [43]. After the weight loss intervention, the AUC of these biomarkers were more sensitive than their fasting values for detecting the beneficial effects of weight loss (Table 2 and Figure 3). It has been suggested that challenge tests can detect more subtle and earlier changes in metabolism than fasting parameters [[44], [45], [46], [47]] mostly based on studies where insulin sensitivity decreased either because of weight gain (energy excess) or in observational studies comparing different degrees of insulin sensitivity. A careful evaluation of the OGTT during a 4-wk weight gain study revealed that metabolic parameters (glucose, triglycerides, IL-6, and IL-18) remained stable in the fasting state, whereas the postprandial responses were different, revealing metabolic derailing, especially when insulin, C-peptide, and glucagon were considered [47]. In the NutriTech study as with recently published results, only subtle changes in response to the dietary challenges after weight loss were observed [39]. Possibly, the inclusion of solely healthy subjects (as assessed by HbA1c, fasting glucose, and insulin at recruitment) had an impact on the magnitude of metabolic improvements after weight loss, and in turn the assessment of phenotypic flexibility. Previously in the NutriTech study, we reported the presence of metabotypes, and we observed that 2 distinct subgroups had varying levels of phenotypic flexibility at baseline and exhibited different responses to the weight loss intervention [46]. The subgroup with reduced phenotypic flexibility at baseline was the only one to demonstrate improvements in multiple health-related biomarkers due to the weight loss intervention [46].

The weight loss intervention had its most striking effect on an individual level in the WLG, as demonstrated by the correlations between changes in various parameters, in particular, changes where fat distribution showed strong associations with changes in multiple metabolic parameters (FIGURE 2, FIGURE 4). This approach does not necessitate a control group or a homogeneous population but rather benefits from a heterogeneous population. With the emergence of extensive phenotyping, homogeneous grouping becomes challenging as stratification on primary objectives may lead to significant variations in several other intervention-related outcomes. Multi-omics studies fully utilize correlational analyses to determine the “connectivity” of the biological processes [48]. Our findings suggest that broadening inclusion criteria would enhance the strength of the correlational analyses while reducing the strength of the group-wise comparisons.

Although the number of subjects was limited, an analysis aimed at identifying a metabolic signature associated with individual susceptibility to improved metabolism following a weight loss intervention successfully identified plasma metabolites during the OGTT and MMTT that served this purpose, as demonstrated by a PLS-DA (Table 2). The BCAA-derived intermediate 2-methyl-butyryl-carnitine is among the top discriminating metabolites, displaying plasma concentrations at least 30% higher at the baseline in subjects who displayed improved insulin sensitivity after weight loss. This metabolite as well as the BCAA precursors are often reported with higher concentrations in the plasma of obese or insulin-resistant subjects [28,29]. Shah et al. [49] reported similar findings with a linear relationship between the level of plasma BCAA at baseline and improved insulin sensitivity induced by weight loss [50]. Moreover, a novel observation in our study was that plasma concentration of DCA was at least 100% higher throughout the OGTT and MMTT in participants with improved insulin sensitivity after weight loss. In these subjects, glycodeoxycholic acid (GDCA) levels were also elevated although the concentration of TUDCA was significantly lower in comparison with individuals that did not experience improved insulin sensitivity, despite having lost a similar amount of BW (Figure 5). As part of the results from the NutriTech study, our group recently described a higher concentration of DCA and GDCA during the OGTT among individuals with improved glucose homeostasis [51]. Plasma urea concentration was also among the variables most implicated in separating the 2 groups in the PLS-DA model. Urea levels were 15–20% higher in participants with improved insulin sensitivity during the weight loss intervention. It is known since the 1950s that the urease activity of gut microbiota plays an important role in nitrogen homeostasis with the microbiota consuming ∼15%–30% of all urea produced by the liver [52]. The participation of intestinal microbiota in the metabolism of BA is also known for decades, being the microbial community responsible to produce secondary BA, such as those that are elevated in the plasma of individuals with improved insulin sensitivity after weight loss. Thus, our results suggest an involvement of gut microbiota in the differential composition of the plasma metabolome among individuals with different responses to the weight loss intervention, generating a hypothesis that remains open for investigation. As a counterargument, diet could also be a determinant of urea concentration in plasma, although our results suggest that both groups of volunteers had similar protein intake before and during the intervention (Figure 6).

The analysis of dietary diaries suggests that the group with improved insulin sensitivity following weight loss was also one with a more positive change in diet quality during the intervention. This group demonstrated a larger increase in fiber intake and a pronounced reduction in the intake of saturated fat, which was not observed in the group with any improvement in insulin sensitivity (Figure 6). Although this may partly explain the better metabolic outcome in these subjects, the data set does not support the claim that better dietary quality was associated with metabolic outcomes.

As with all studies investigating the relationship between dietary change and metabolism in overweight and obese cohorts, self-reported intake should be cautiously interpreted. Although we estimated compliance with the energy restrictions in the WLG to be good, the urine metabolomics analyses suggest a careful interpretation of the diet quality. We observed a small but significant improvement in dietary quality in the WLG, which was not seen in the WMG. This may explain why the difference in metabolic outcomes was less clear than expected.

In conclusion, we demonstrate that moderate weight loss affected some metabolic parameters with a large interindividual variation. We provide novel evidence that a dietary challenge may be a sensitive tool to detect subtle changes in the metabolism induced by weight loss, offering an advantage to conventional plasma analysis after overnight fasting. OGTT was more sensitive than MMTT in detecting changes in markers of insulin sensitivity. We also observed that some participants exhibited improved insulin sensitivity whereas others did not, despite similar decreases in BW. A metabolic signature for such predisposition was proposed, suggesting that extensive phenotyping and dietary challenges are useful tools for the development of personalized nutrition. Full interpretation of complex nutritional data at an individual level, such as that presented in this study, needs new methodologies of interpreting and visualizing such as those offered by machine learning, AI, and modeling.

## Acknowledgments

This project was supported by the Imperial NIHR Clinical Research Facility at Imperial College Healthcare NHS Trust. The NutriTech consortium is very thankful to Biocrates Life Sciences (Innsbruck, Austria) for the donation of the kits for LC-MS/MS-based analysis of plasma metabolites such as amino acids, glycerophospholipids, and acylcarnitines.

### Author contributions

The authors’ responsibilities were as follows – GF, BO, HD, JB, ELT, LB, SW, DI, CD: designed the research program. All authors: contributed to the research leading to the findings reported here; GF, MR, JF: lead the writing of the manuscript with input from all authors. MR, JF, ELT, SW, LAA, LB, CAD, TEG, HD, IGP, JMP, DGI, JDB, BVO, GF: read and commented on the final version of the manuscript.

### Conflict of interest

IG-P, JMP, and GF hold shares in Melico Sciences Ltd, and IG-P and GF are directors in the company. Melico has developed a quantitative method of assessing dietary intake using the same type of technology (NMR) as was used here to analyze urine samples. Melico was not involved in, or benefits from, this study. TEG is CEO and stock owner, and CAD is the consultant, board member, and stock owner in the analytical laboratory Vitas Ltd, Oslo, Norway.

### Funding

This study was funded by the EU 7th Framework Grant number KBBE-20115-289511.

The study is registered at ClinicalTrials.gov with the registration ID NCT01684917 and can be viewed at https://clinicaltrials.gov/ct2/show/NCT01684917.

### Data Availability

Data described in the manuscript, code book, and analytic code will be made available upon request to the corresponding author.
