# Supplementary material for: Diet-induced Weight Loss and Phenotypic Flexibility Among Healthy Overweight Adults: A Randomized Trial
Source: Am J Clin Nutr. 2023 Aug 28;118(3):591–604. doi: 10.1016/j.ajcnut.2023.07.002 (PMC10517213; doi:10.1016/j.ajcnut.2023.07.002)
Supplement: Multimedia component 1 [file mmc1.docx]

**Diet-induced weight loss and phenotypic flexibility among healthy overweight subjects. A randomised trial. Mi**lena Rundle etal

**Supplementary methods**

**Recruitment of volunteers**

We contacted 22,000 individuals by post and 2,900 expressed interest in participating. Screening via telephone reduced this number to 210, and 191 were screened for the energy restriction study. The recruitment numbers and flow in each part of the study are shown in the consort diagram (Supplemental Figure 1) with demographic details presented in Table 1. In total 78 participants underwent MR imaging at baseline and 68 at follow-up. Data are presented from the 68 patricipants (36 males, 32 female) completing the intervention. Ten subjects did not complete the intervention, 9 (6 males, 3 female) withdrew before their follow-up scan, one (woman) was unable to complete her follow-up scan, so scanning was terminated. Similar numbers dropped out of both the control and the intervention groups. Of the 68 participants completing the study, 31 were in the control group, whereas 37 were in the weight loss group.

**Screening visit**

Following initial phone screening, participants attended a health-screening visit at the research facility. The assessment included measurements of height, weight, body fat percentage by bioelectric impedance, blood pressure, a 12-lead electrocardiogram , blood sample for following analyses of glucose, insulin, glycated haemoglobin (HbA1c), full blood count , liver function test , urea and electrolyte test (U+E) for kidney function, and lipids. Details of medical history, medications and lifestyle were collected. Current dieters were not included’ Participants currently on medications not interfering with metabolism (otherwise excluded) and dietary supplements were asked to continue the use throughout the study. Abnormal results were reported to the GP for follow-up. Inclusion and exclusion criteria are presented in Supplemental Table 1.

**Anthropometry**

Body weight (kg) was acquired using a Seca scale (Vogel & Halke Hamburg, Germany). Height (m) was recorded using a stadiometer (Invicta Plastics Ltd., Leicester, UK). Waist and hip circumferences (cm) were measured by an experienced observer. Waist circumference was measured at the (WHO recommended) midpoint between the distal border of the lowest rib and the superior border of the iliac crest.

**Assessment week**

We performed an extensive series of assessments prior to the start of the energy restriction and then at week 12 as presented in Supplemental Figure 2.

Participants were instructed to avoid alcohol and strenuous exercise prior to each study visit. Visits started at 8:30am following a 12 h fast, when participants were daily residents at the Clinical Research Facility.

Day 1 Fasting

Day 1 of the assessment week provided fasting measures. All anthropometric measurements were collected, followed by resting energy expenditure. MRI was performed as previously described ([1](#_ENREF_1)). Blood samples were collected by a single venepuncture.

Day 2 OGTT

An oral glucose tolerance test was performed giving 75 g of glucose in 250 mL of water ([2](#_ENREF_2)). Following the insertion of a cannula, a fasting blood samples were taken at 9:00 am and 15, 30, 60, 90, 120, and 240 min after consumption of the glucose drink. After the last sample participants were given lunch of their choice and discharged home.

Day 3 MMTT

A nutritional stress test was performed using a mixed meal tolerance test according to a method previously established ([2](#_ENREF_2)). It included 75g of glucose, 60g of palm oil and 20g of protein (Protifar, Nutricia) mixed with 320 g of water. Following the fasting sample, the mixed meal tolerance test was consumed over 5 min, subsequent blood samples were taken at 60, 120, 240, 360 and 480 min from the start of drinking. After collection of the last sample, participants were given lunch of their choice and discharged home.

**Dietary assessment**

Habitual food intake was assessed in week 1 by a 7-day food diary and again in week 13 to check for compliance to the prescribed diet. Subjects were instructed to estimate portion sizes using household measures such as cups and tablespoons. Brand names were also given where appropriate. Food diaries were analyzed using dietary analysis software Diet Plan 6.70 software (Forestfield Software Ltd, 1991-2012) for complete macro- and micronutrients profile; a food portion size book ([4](#_ENREF_4)) was used where food quantities were omitted from the diary.

**Classical biochemistry**

Blood samples were centrifuged at 1800 rpm, and plasma and serum samples were stored at -80ºC. Serum insulin level was measured by radioimmunoassay using a Millipore Human Specific Insulin RIA Kit (Millipore Corporation) accordingly to the manufacturer’s instructions.

Serum glucose level was measured by an enzymatic method using an Abbott Architect ci8200 analyzer.

The gut hormones PYY and GLP-1 in plasma were measured using an in-house radioimmunoassay with a previously established method ([5](#_ENREF_5)).

Albumin, ammonia, urea, creatinine, aspartate aminotransferase, total cholesterol, HDL-cholesterol, LDL-cholesterol, and uric acid levels were measured using standard methodology, according to the manufacturer’s instructions.

Non-esterified fatty acids (NEFA) were quantified with an enzymatic assay (Wako Diagnostics, Mountain View, CA, USA).

Leptin levels in blood plasma were assayed by a sandwich enzyme immunoassay (ELISA) using plates coated with polyclonal anti-human leptin antibody and a horseradish peroxidase conjugate of the antibody (HUMAN LEPTIN ELISA, cat no: RD191001100, BioVendor – Laboratorní medicína AS, Czech Republic).

Gamma-glutamyltransferase and triglycerides in blood plasma were assayed on Olympus AU400 automated analyzer using commercially available kit of Beckman Coulter International SA, Switzerland (by kinetic UV method for GGT (gamma-glutamyl transferase), Reagent, cat. No. OSR6120), and by enzyme colorimetric method for triglycerides, TRIG Reagent, cat. No. OSR61118).

**Energy expenditure**

Resting energy expenditure was assessed by indirect calorimetry (GEM, UK) on day 1 of each assessment week in a fasted state. Participants were asked to rest for 15 min in a semi-recumbent position before the measurement. Calibration and measurement were collected using a well-established method ([6](#_ENREF_6)). A large transparent canopy was placed over the head and thorax. Once the carbon dioxide content of the air entering the chamber stabilized, participants were asked to stay still for 20 min during gas collection. Resting metabolic rate (RMR) and respiratory quotient (RQ) were obtained. Assessment of physical activity was performed in line with diet diaries. Participants were asked to wear an accelerometer (BodyMedia SenseWear, Pittsburgh, USA) on the non-dominant arm for 7 days in week 1 and week 13. Final value was presented as day average in min. Total energy expenditure (TEE) was calculated as Resting Energy Expenditure (kcal) x Activity (METs). TEE represented the basis for dietary intervention in the maintenance group. The energy restricted group followed a 20 % energy restriction diet; thus, 20 % of energy were taken off their TEE to achieve a five % reduction in weight for 12 weeks.

**VO_2_ max**

Participants performed an incremental exercise test on an ergometer cycle (Cosmed, Italy). Cycling started at 100 Watts (W) with the workload increasing by 25 W every 3 min until volitional exhaustion. Maximum oxygen uptake (VO_2_max), maximum aerobic power output (Wmax), and maximum heart rate (HRmax) were calculated.

**Whole-body MRI**

All MRI studies were performed on a 1.5T multinuclear scanner (Achieva, Philips Medical Systems, Best, Netherlands)([1](#_ENREF_1)). Briefly, images were acquired using whole body axial T_1_-weighted spin echo sequence, a body coil, and no respiratory gating (typical parameters: repetition time (TR) 560 ms; echo time (TE) 18 ms; slice thickness 10 mm; interslice gap 10 mm; flip angle 90 degrees; number of excitations 1).

Subjects were positioned in the magnet in a prone position with their arms straight above their head and scanned from their fingertips to their toes. Images were acquired as 9 equal stacks of 12 slices at the isocentre of the magnet. Images were analyzed by Vardis (Vardis Group, London, UK) using SliceOmatic, (Tomovision, Montreal, Canada). Total and regional adipose tissue volumes were recorded in liters including total subcutaneous adipose tissue, which was further divided into abdominal subcutaneous adipose tissue, peripheral subcutaneous adipose tissue, and total internal adipose tissue, which was subdivided into visceral adipose tissue and non-abdominal internal adipose tissue.

**^1^H MRS of liver**: Spectra were acquired using a PRESS sequence without water suppression (typical parameters: TR 1500 ms; TE 135 ms; voxel size 20x20x20 mm; flip angle 90 degrees, number of excitations 64). Transverse images of the liver were used to ensure the positioning of the voxel, which was placed in an area of the liver avoiding the gall bladder, adipose tissue, and major blood vessels. Spectra were analyzed using the AMARES (advanced method for accurate, robust, and efficient spectral fitting) algorithm included in the MRUI software package. Peak areas for all resonances were obtained and lipid resonances were quantified with reference to water after correcting for T_1_ and T_2_  ([7](#_ENREF_7)).

**^1^H MRS of muscle:** Spectra were obtained from *m. soleus* and *m. tibialis* of the left calf, using a PRESS sequence as previously described with typical parameters: TR 1500 ms; TE 135 ms; voxel size 20x20x20 mm; flip angle 90 degrees, number of excitations 64. Transverse images of the calf were used to ensure the positioning of the voxel in an area avoiding visible streaky fat and major blood vessels. Spectra were analyzed using AMARES, lipid resonances quantified with reference to Cr_tot_ after correcting for T_1_ and T_2_ ([9](#_ENREF_9))_._

**MRI of pancreas**: Single slice multi-echo (ME) was performed through the pancreas during a single breath hold of 15 seconds. Typical parameters FOV 400*300 mm, TR150 ms, 10 mm slice thickness; 20 TEs starting at 1.15 ms with a spacing of 1.15 ms producing alternate in and out of phase images. Analysis was performed using MATLAB (MathWorks Inc, MA, USA) to generate measurements of pancreatic fat ([8](#_ENREF_8)).

**Supplemental figures**

**Supplemental Figure 1.** Consort diagram showing recruitment to the weight loss intervention of the NutriTech study.

Excluded (n= 61)

♦  Not meeting inclusion criteria (n= 46)

♦  Declined to participate (n=15)

Intervention

Weight loss

Control

Weight maintenance

Randomized (n= 130)

## Contacted

Contacted but post (n= 22,600)

## Follow-Up

Analysed (n= 32)

## Analysis

Analysed (n= 40)

Lost to follow-up (give reasons) (n= 15)

Discontinued intervention (give reasons) (n= 2)

Lost to follow-up (give reasons) (n= 21)

Discontinued intervention (give reasons) (n=5)

## Enrollment

Allocated to intervention (n= 65)

♦ Received allocated intervention (n=58)

♦ Did not receive allocated intervention (give reasons) (n= 7 )

## Allocation

Allocated to intervention (n= 64)

♦ Received allocated intervention (n=57)

♦ Did not receive allocated intervention (give reasons) (n=7 )

Assessed for eligibility (n=191)

**Supplemental Figure 2.** Diagram showing the Magnetic Resonance Imaging (MRI) phenotyping employed in the NutriTech study. A) coronal pilot MRI scan used for planning subsequent acquisitions to illustrate positioning of further scans; B) whole body MRI data comprising of 100-113 (depending on height) T1-weighted images acquired in an axial plane covering the entire body from the fingertips to toes; C) T1-weighted images acquired at mid-calf level, showing the position of PRESS spectroscopy voxels in the m. soleus (pink) and m. tibilais (red), and a corresponding proton magnetic resonance spectroscopy (1H MRS) spectrum obtained from m. soleus; D) Multi-echo MRI images (ME MRI) and corresponding analyses acquired for pancreas, and E) liver; F) T1-weighted images acquired at the level of the liver, showing the position of PRESS spectroscopy voxel and corresponding 1H MR spectrum.

**F**


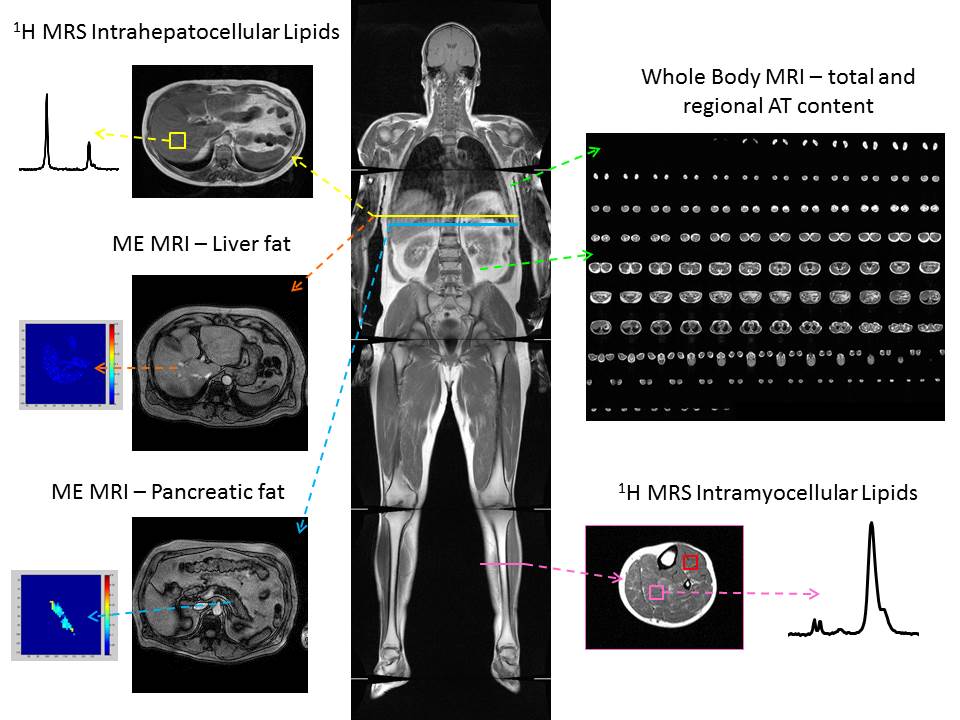


**D**

**E**

**C**

**B**

**A**

**Supplemental Table 1**. Inclusion and exclusion criteria for the energy restriction study

| **Inclusion criteria** |
| --- |
| Healthy overweight and obese participants, BMI of 25-35 kg/m^2^, 50-65 years of age |
| **Exclusion criteria** |
| - Weight change of ≥ 3kg in the preceding 3 months - Current smokers - Substance abuse - Excess alcohol intake - Pregnancy - Diabetes - Cardiovascular disease - Cancer - Gastrointestinal disease e.g. inflammatory bowel disease or irritable bowel syndrome - Kidney disease - Liver disease - Pancreatitis - Use of medications likely to interfere with energy metabolism, appetite and hormonal regulation including anti-inflammatory drugs or steroids, antibiotics, androgens, phenytoin, erythromycin, and thyroid hormones - Having metallic or magnetic implants such as pacemakers - Claustrophobia |

**Supplementary figure 3: Summary of weight loss of the volunteers. A. comparison of weight loss in the weight maintenance and weight loss group compared to estimated weight loss. B. the individual weigh lose over 12 weeks.**

**a.**

**b.**

**
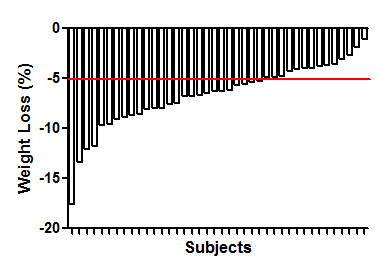
**

**Supplemental Table 2**. **Variables with the highest VIP values as indicated by the PLS-DA model considering data from metabolomics analysis of plasma collected during the OGTT and MMTT and body composition.** The analysis compared individuals exhibiting improved insulin sensitivity with those subjects who did not display improved insulin sensitivity after weight loss. Variables in red (negative loading scores) were associated with the participants who did not improve insulin sensitivity.

**Table 2 – continuation**

**Supplemental references**

1. Thomas EL, Saeed N, Hajnal JV, Brynes A, Goldstone AP, Frost G, et al. Magnetic resonance imaging of total body fat. Journal of applied physiology (Bethesda, Md : 1985). 1998;85(5):1778-85.

2. Stroeve JH, Wietmarschen H, Kremer BH, Ommen B, Wopereis S. Phenotypic flexibility as a measure of health: the optimal nutritional stress response test. Genes & nutrition. 2015;10(3):1.

3. Li Y, Lee S, Langleite T, Norheim F, Pourteymour S, Jensen J, et al. Subsarcolemmal lipid droplet responses to a combined endurance and strength exercise intervention. Physiological reports. 2014;2(11):e12187.

4. Crawley Helen AM, and Sejal Patel. Food Portion Sizes (Maff Handbook). London: TSO; 1993.

5. Kreymann B, Ghatei M, Williams G, Bloom S. Glucagon-like peptide-1 7-36: a physiological incretin in man. The Lancet. 1987;330(8571):1300-4.

6. Tan TM, Field BC, McCullough KA, Troke RC, Chambers ES, Salem V, et al. Coadministration of glucagon-like peptide-1 during glucagon infusion in humans results in increased energy expenditure and amelioration of hyperglycemia. Diabetes. 2013;62(4):1131-8.

7. Thomas EL, Hamilton G, Patel N, O’dwyer R, Doré CJ, Goldin RD, et al. Hepatic triglyceride content and its relation to body adiposity: a magnetic resonance imaging and proton magnetic resonance spectroscopy study. Gut. 2005;54(1):122-7.

8. Thomas EL, Fitzpatrick JA, Malik SJ, Taylor-Robinson SD, Bell JD. Whole body fat: content and distribution. Progress in nuclear magnetic resonance spectroscopy. 2013;73:56-80.

9. Rico-Sanz J, Thomas EL, Jenkinson G, Mierisova S, Iles R, Bell JD. Diversity in levels of intracellular total creatine and triglycerides in human skeletal muscles observed by (1)H-MRS. Journal of applied physiology (Bethesda, Md : 1985). 1999;87(6):2068-72.
